# Defective Molecular Timer in the Absence of Nucleotides Leads to Inefficient Caspase Activation

**DOI:** 10.1371/journal.pone.0016379

**Published:** 2011-01-27

**Authors:** Honghao Zhang, Raghu Gogada, Neelu Yadav, Ravi K. Lella, Mark Badeaux, Mary Ayres, Varsha Gandhi, Dean G. Tang, Dhyan Chandra

**Affiliations:** 1 Department of Pharmacology and Therapeutics, Roswell Park Cancer Institute, Buffalo, New York, United States of America; 2 Department of Molecular Carcinogenesis, The University of Texas MD Anderson Cancer Center, Smithville, Texas, United States of America; 3 Department of Experimental Therapeutics, The University of Texas MD Anderson Cancer Center, Houston, Texas, United States of America; University of Dayton, United States of America

## Abstract

In the intrinsic death pathway, cytochrome C (CC) released from mitochondria to the cytosol triggers Apaf-1 apoptosome formation and subsequent caspase activation. This process can be recapitulated using recombinant Apaf-1 and CC in the presence of nucleotides ATP or dATP [(d)ATP] or using fresh cytosol and CC without the need of exogenous nucleotides. Surprisingly, we found that stored cytosols failed to support CC-initiated caspase activation. Storage of cytosols at different temperatures led to the loss of all (deoxy)nucleotides including (d)ATP. Addition of (d)ATP to such stored cytosols partially restored CC-initiated caspase activation. Nevertheless, CC could not induce complete caspase-9/3 activation in stored cytosols, even with the addition of (d)ATP, despite robust Apaf-1 oligomerization. The Apaf-1 apoptosome, which functions as a proteolytic-based molecular timer appeared to be defective as auto-processing of recruited procaspase-9 was inhibited. Far Western analysis revealed that procaspase-9 directly interacted with Apaf-1 and this interaction was reduced in the presence of physiological levels of ATP. Co-incubation of recombinant Apaf-1 and procaspase-9 prior to CC and ATP addition inhibited CC-induced caspase activity. These findings suggest that in the absence of nucleotide such as ATP, direct association of procaspase-9 with Apaf-1 leads to defective molecular timer, and thus, inhibits apoptosome-mediated caspase activation. Altogether, our results provide novel insight on nucleotide regulation of apoptosome.

## Introduction

Caspases, the core enzymes responsible for executing apoptotic cell death, are synthesized as inactive zymogens and divided into initiator (caspase-2, -8, -9, and –10) and effector or executioner (caspase-3, -6, and –7) caspases. Active initiator caspases generated in response to apoptosis signals induce intrachain cleavage of effector caspases, which undergo reorganization of active site loops to become active [1]. Activation of initiator caspases such as caspase-9 requires the adaptor protein Apaf-1 [2], which contains caspase-recruitment domain (CARD), nucleotide binding and oligomerization domain (NOD), and WD40 repeats for CC interaction. The released CC from mitochondria binds to and induces oligomerization of Apaf-1 to form the ‘apoptosome’, a heptameric complex with molecular masses of ∼700–1400 kDa [3], [4], [5]. Procaspase-9 subsequently becomes activated within the apoptosome either involving proximity-induced dimerization or “induced conformational changes” [1].

Among various factors that regulate apoptosome formation and caspase activation, (d)ATP plays a critical role. Cell-free and recombinant protein reconstitution experiments have demonstrated that (d)ATP initiates Apaf-1 oligomerization following Apaf-1 binding to CC [5], [6], [7]. Truncated Apaf-1, i.e., Apaf-591, which lacks the WD-40 repeats but retains the NOD, binds to ADP molecule, which locks Apaf-1 in a conformationally inactive state [8]. Full-length Apaf-1 is capable of binding one molecule of dATP. CC binding to Apaf-1 induces hydrolysis of dATP to dADP coupled with exchange for dATP to initiate apoptosome assembly [9], [10]. Thus, nucleotide binding and exchange are critical for the regulation of apoptosome formation and caspase activation. It is of interest that once functional apoptosome is assembled, recruited procaspase-9 is processed within the apoptosome. The processed caspase-9 fragment possesses lower affinity for apoptosome and is continuously replaced by procaspase-9. Therefore, the Apaf-1 apoptosome functions as proteolytic-based “molecular timer”, wherein the autoprocessing of procaspase-9 starts the timer and intracellular levels of procaspase-9 sets the overall duration of the timer [11], [12].

Most mammalian cells have an intracellular ATP and nucleotide pool in millimolar range [13], [14], [15], [16], [17]. For example, the cytoplasmic levels of ATP alone can be as high as 3–8 mM [13], [14], [15], [16], which explains our recent observations that freshly purified cytosol does not require exogenous (d)ATP to initiate apoptosome assembly and caspase activation by CC [16]. Here we report that cytosols stored at low temperatures fail to fully support the CC-mediated caspase activation. Loss of (d)ATP causes stable association of procaspase-9 with the apoptosome. Altogether, degradation of nucleotides leads to dysfunctional molecular timer, and thus, blocking sustained activation of caspase-9 on the apoptosome.

## Results

### dATP is required for CC-initiated caspase activation by recombinant Apaf-1 but not by freshly purified cytosol

**Figure 1:**
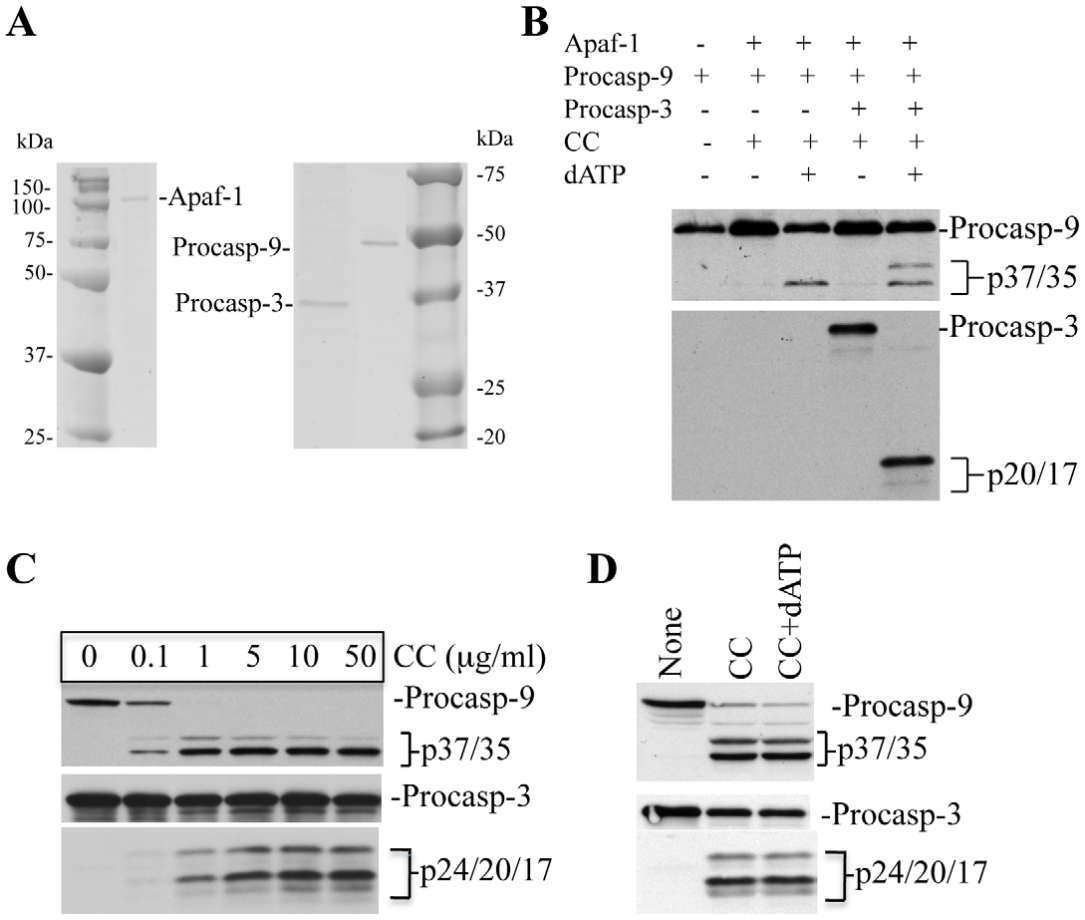
Fresh cytosol does not require exogenous nucleotides for CC-initiated caspase activation. **A**, Recombinant Apaf-1, procaspase-9 (procasp-9), or procaspase-3 (procasp-3) was expressed in insect cells and subsequently purified. These proteins were separated on SDS-PAGE and stained with Coomassie blue. **B,** Reconstitution experiments were performed to test the activity of purified proteins by using recombinant Apaf-1 (165 ng), procaspase-9 (130 ng), and procaspase-3 (100 ng) with or without CC (15 µg/ml) and dATP (200 µM) in a total reaction mixture of 30 µl. At the end of incubation at 30°C for 120 min, aliquots were subjected to Western blotting for caspase-9 and caspase-3. **C,** Fresh GM701 cytosol (250 µg) incubated for 150 min at 37°C with increasing amount of CC was used in Western blotting for caspase-9 and caspase-3. **D**, Fresh HeLa cytosol incubated with CC (15 µg/ml) in the absence or presence of dATP (200 µM) was subjected to Western blotting for caspase-9 and caspase-3. Procasp-9, procaspase-9; procasp-3, procaspase-3; CC, cytochrome c.

Exogenous (d)ATP is required for caspase activation in reconstitution experiments using recombinant proteins or purified cytosol [3], [4], [5], [7]. On the other hand, we recently found that in reconstitution experiments using freshly purified cytosol, exogenous (d)ATP was not required for CC-initiated caspase activation [16], [18]. To clarify this seeming ‘discrepancy’, we first purified recombinant Apaf-1, procaspase-9, and procaspase-3 from insect cells (Sf9) (Fig. 1A) and used theses purified proteins in CC-initiated caspase assays (Fig. 1B). The results showed that (d)ATP was absolutely required for Apaf-1/CC-initiated caspase activation (Fig. 1B, and data not shown). For example, in the absence of dATP, there was no cleavage of procaspase-9 or procaspase-3 (Fig. 1B). However, in the presence of dATP, the p35 caspase-9 was generated through Apaf-1-dependent autocatalytic cleavage of procaspase-9 at Asp^315^ (Fig. 1B). In the presence of both dATP and procaspase-3, the p37 caspase-9 was also generated (Fig. 1B) as a result of caspase-3-mediated cleavage of procaspase-9 at Asp^330^ [16], [19]. Procaspase-3 was cleaved by activated caspase-9 to generate active p20/p17 fragments (Fig. 1B).

In contrast to the above reconstitution experiments using purified proteins, CC, in a concentration-dependent manner, initiated caspase-9 and -3 cleavage in fresh cytosol purified from GM701 cells without requiring exogenous dATP (Fig. 1C). Likewise, similar levels of procaspase-9 and -3 cleavage were observed in fresh HeLa cell cytosol in the absence or presence of dATP (Fig. 1D). Measurements of caspase-3 and caspase-9 activities (data not shown) also revealed that CC initiates caspase activation without the need of exogenous (d)ATP in fresh cytosols from GM701, NHDF (normal human dermal fibroblasts) and HMEC (human mammary epithelial cells) cells. Similar to cell-free reconstitution experiments using fresh cytosols from multiple cells including MDA-MB231, LNCaP, PPC-1, PC3, Du145, NHP, K562, HCT116, and C2F3 (see Materials and Methods for descriptions of cells) also revealed CC-initiated caspase activation without requiring exogenous nucleotides (data not shown) [16].

The above observations suggest that freshly purified cytosols contain sufficient amounts of nucleotides to assist in the CC-initiated caspase activation. In support, direct measurements of (d)NTP levels in 6 tumor cell lines and 3 primary cell strains revealed that these cells contain ATP levels of 1.5–5 mM and possessed an NTP pool of ∼3–7 mM and a dNTP pool of ∼10–80 µM [16].

### Stored cytosols fail to support CC-initiated caspase activation

**Figure 2:**
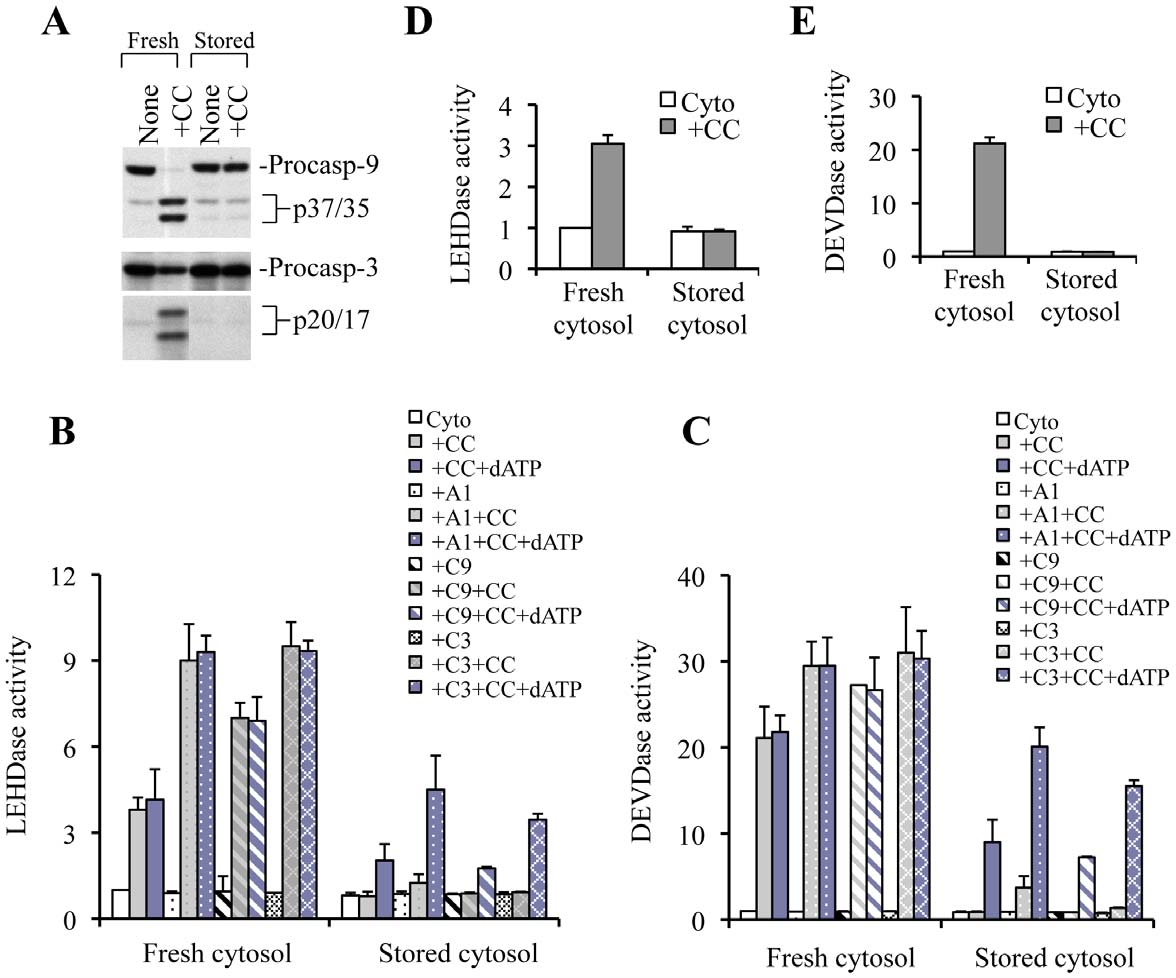
Stored cytosols require exogenous nucleotides to support CC-initiated caspase activation. **A**, GM701 cytosols (250 µg) either freshly purified or stored at 4°C for 5 days were incubated with CC (15 µg/ml) for 150 min at 37°C. At the end of incubation, reaction mixtures were subjected to Western blotting for caspase-9 and caspase-3. **B and C**, GM701 cytosols (250 µg) either freshly purified or stored at 4°C for 5 days were incubated with CC in the absence or presence of dATP (200 µM). Some reaction mixtures were supplemented with recombinant Apaf-1 (A1) or procaspase-9 (C9) or procaspase-3 (C3) to assess if these molecules were inactivated during storage. **D and E**, HCT116 cytosols either freshly purified or stored at 4°C for 5 days were incubated with CC. At the end of incubation, aliquots were used in activity assays for caspase-9 and caspase-3. Procasp-9, procaspase-9; procasp-3, procaspase-3; CC, cytochrome c.

**Figure 3:**
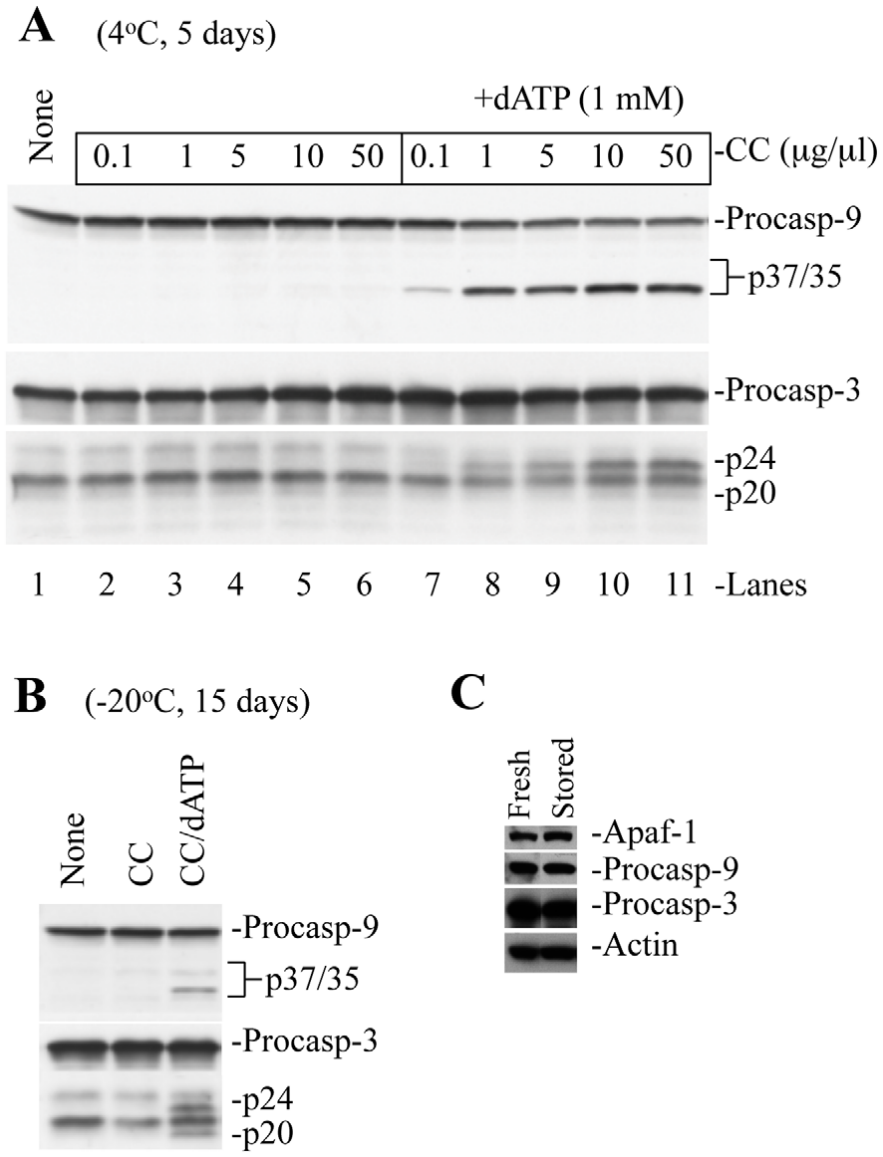
Exogenous dATP restores CC-initiated caspase activation. **A**, GM701 cytosols stored at 4°C for 5 days were incubated with increasing amounts of CC in the absence (lanes 1–6) or presence (lanes 7–11) of dATP (1 mM). **B,** Cytosol stored at −20°C for 15 days were incubated with CC in the absence or presence of ATP (1 mM). At the end of incubation, samples were subjected to Western blotting for caspase-9 or caspase-3. **C,** Cytosols stored at 4°C for 5 days were subjected to Western blotting to detect the levels of indicated proteins. Procasp-9, procaspase-9; Procasp-3, procaspase-3; CC, cytochrome c.

To determine whether cytosol could be stored for later use to investigate the mechanisms of functional apoptosome assembly, we tested the capability of cytosol stored at different temperatures. Much to our surprise, when we performed similar cell-free reconstitution experiments using purified GM701 cytosols stored for 5 days at 4°C, we found that addition of CC alone could not support caspase activation, whereas complete caspase-9/3 processing was observed in fresh cytosol (Fig. 2A; Fig. 3A). Substrate cleavage assays also demonstrated no increase in caspase-9 (Fig. 2B) or caspase-3 (Fig. 2C) activity in stored cytosols. We then performed cell-free reconstitution experiments using either freshly purified cytosol from HCT116 cells or HCT116 cytosol stored at 4°C for 5 days. As shown in Fig. 2D and 2E, stored cytosol failed to support the CC-initiated caspase activation. We then examined GM701 cytosols stored at −20°C or −80°C for different periods of time. We observed that cytosols stored at −20°C for 15 days could not support CC-initiated caspase activation (Fig. 3B). Cytosols stored at −80°C for 1 month supported CC-induced caspase activation, whereas storage of cytosols at −80°C for longer periods of time failed to activate caspases (data not shown).

To examine if storage of cytosol leads to degradation of protein required for apoptosome assembly, we performed Western blotting to detect their levels and demonstrated that stored cytosols possess similar levels of Apaf-1, procaspase-9, and procaspase-3 (Fig. 3C), suggesting that failure of stored cytosols to support the CC-initiated caspase activation is not caused by quantitative differences in these three main constituents of the apoptosome. To determine whether during storage of cytosol, the main components of apoptosome might have undergone conformational changes, and therefore, become functionally inactive, we performed reconstitution experiments using stored cytosols by adding CC along with recombinant Apaf-1 or procaspase-9 or procaspase-3. We demonstrated that addition of CC in the presence/absence of each individual recombinant protein did not significantly alter caspase-9 or caspase-3 activation (Fig. 2B–C). These experiments indicate that failure of stored cytosols to support the CC-initiated caspase activation does not seem to be associated with degradation/inactivation of the core protein components of the Apaf-1-apoptosome.

### (Deoxy)nucleotides become degraded and exogenous dATP partially restores CC-mediated caspase activation in stored cytosols

Considering the absolute requirement of (d)ATP in Apaf-1-mediated caspase activation (Fig. 1B), we reasoned that failure of stored cytosols to support CC-initiated caspase activation might be associated with degradation of nucleotides including (d)ATP. To test this possibility, purified cytosols from GM701 cells were kept at 4°C, −20°C, or −80°C for various periods of time followed by measurement of (deoxy)nucleotides concentrations. As shown in Table 1 and 2, storage at 4°C for 2 days led to >90% loss and storage for ≥4 days resulted in nearly complete degradation of all (deoxy)nucleotides. Similarly, storage at −20°C for 4 days led to ≥40% loss and for 15 days to ∼85% loss of most (deoxy)nucleotides including ATP (Table 1 and 2). dATP appeared to be slightly more stable as 15-day storage resulted in ∼60% of its loss (Table 2). Even storage at −80°C for 2 months led to nearly 50% loss of all (deoxy)nucleotides (Table 1 and 2). These results indicate that storage of cytosol samples at low temperature, as routinely practiced in laboratory research, may lead to rapid degradation of (deoxy)nucleotides.

**Table 1:** Changes in nucleotide levels during storage at different temperatures*.

Storage	Storage	Nucleotide levels (%)			
temperature	time	ATP	UTP	GTP	CTP
Fresh	-	35.8 (100)	11.5 (100)	10.0 (100)	2.4 (100)
cytosol					
−80°C	1 month	36.1 (101)	12.1 (105)	10.4 (104)	2.4 (104)
	2 months	18.8 (53)	6.2 (54)	5.3 (53)	1.1 (46)
−20°C	4 days	20.2 (56)	7.2 (62)	6.2 (62)	1.1 (46)
	8 days	14.1 (39)	5.3 (46)	4.6 (46)	0.7 (29)
	15 days	5.5 (15)	1.9 (17)	1.6 (16)	0.3 (12)
4°C	2 days	2.4 (7)	0.9 (8)	0.8 (8)	0.0 (0)
	4 days	1.1 (3)	0.1 (1)	0.2 (2)	0.0 (0)
	8 days	0.7 (2)	0.1 (1)	0.2 (2)	0.0 (0)

*Cytosols obtained from GM701 cells were stored at different temperatures for the time intervals indicated and then nucleotides levels were measured using HPLC. Values represent the mean (nmoles/mg protein) derived from two independent measurements (values in the parentheses represent percentage changes compared to the fresh cytosol).

**Table 2:** Changes in deoxynucleotide levels during storage at different temperatures*.

Storage	Storage	Deoxynucleotide levels (%)			
temperature	time	dATP	dTTP	dCTP	dGTP
Fresh	-	0.103 (100)	0.547 (100)	0.097 (100)	0.027 (100)
cytosol					
−80°C	1 month	0.090 (87)	0.483 (88)	0.090 (93)	0.023 (85)
	2 months	0.043 (42)	0.282 (52)	0.045 (46)	0.012 (44)
−20°C	4 days	0.057 (55)	0.330 (60)	0.020 (21)	0.010 (37)
	8 days	0.057 (55)	0.253 (46)	0.010 (10)	0.007 (26)
	15 days	0.037 (36)	0.090 (16)	0.000 (0)	0.000 (0)
4°C	2 days	0.003 (3)	0.040 (7)	0.003 (3)	0.000 (0)
	4 days	0.000 (0)	0.007 (1)	0.000 (0)	0.000 (0)
	8 days	0.000 (0)	0.007 (1)	0.000 (0)	0.003 (11)

*Cytosols obtained from GM701 cells were stored at different temperatures for the time intervals indicated and then deoxynucleotides levels were measured using HPLC. Values represent the mean (nmoles/mg protein) derived from two independent measurements (values in the parentheses represent percentage changes compared to the fresh cytosol).

To determine whether loss of (d)ATP might be responsible for the failure of stored cytosols to support the CC-mediated caspase activation, we added dATP back to the cytosols stored at 4°C for 5 days (Fig. 3A) or at −20°C for 15 days (Fig. 3B), and in every case, we observed a CC concentration-dependent generation of p35 caspase-9 and p24/p20 caspase-3 bands. Substrate cleavage assays for caspase-9 (Fig. 2B) and caspase-3 (Fig. 2C) also showed partial restoration of their activity by addition of exogenous dATP. Similar results were obtained using ATP in the reconstitution experiments (data not shown).

Surprisingly, dATP addition did not result in full caspase activation to the same level as observed in fresh cytosols (Fig. 2B–C). Furthermore, increasing CC to ≥10 µg/ml did not enhance caspase cleavage (Fig. 3A, lanes 10 and 11). Most strikingly, we observed that dATP addition to stored cytosols resulted in prominent generation of the p35 caspase-9 band without the p37 band (Fig. 3A and B). Since the p35 caspase-9 is generated through Apaf-1-dependent cleavage of procaspase-9 at Asp^315^ and the p37 caspase-9 results from caspase-3-mediated cleavage of procaspase-9 at Asp^330^ [16], [19], these observations suggest that exogenous dATP appeared to have initiated relatively normal apoptosome activation of procaspase-9 in stored cytosols but caspase-3 mediated caspase-9 cleavage/activation was inhibited. It is known that the p24 fragment of caspase-3 has a high affinity for XIAP and further processing to mature p20/p17 fragments is required for full caspase activity [4], [20], [21], [22]. Interestingly, majority of dATP/CC-induced processing of caspase-3 in cytosols stored at 4°C for 5 days (Fig. 3A) or at −20°C for 15 days (Fig. 3B) was p24, suggesting that caspase-3 activation by p35 caspase-9 was incomplete, which could be responsible for a lack of generation of the p37 caspase-9.

### Stored cytosols support CC-initiated Apaf-1 oligomerization and procaspase-9 recruitment but display defective caspase-9 activation in the apoptosome

**Figure 4:**
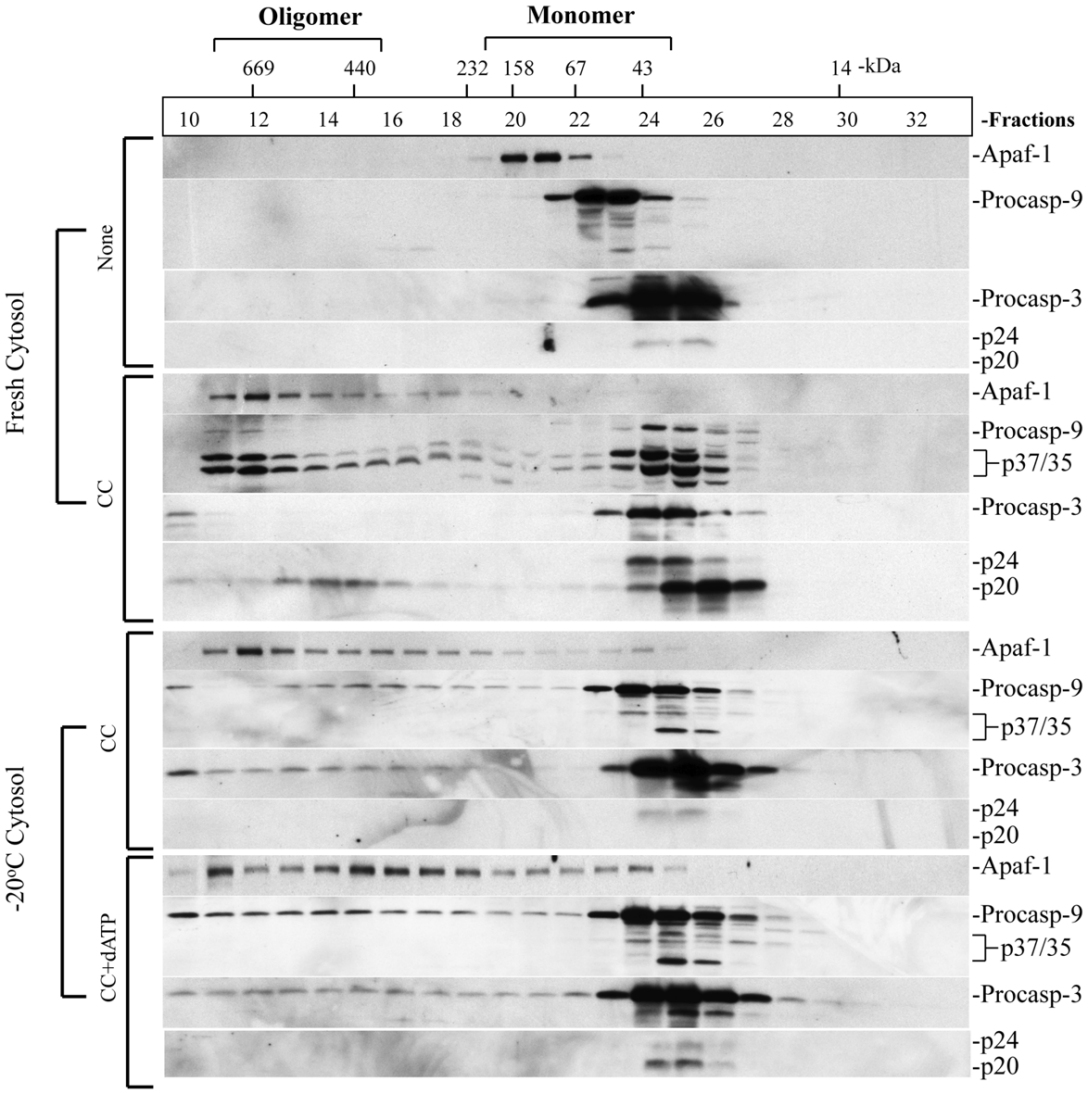
Defective apoptosome formation and caspase activation in stored cytosols. GM701 cytosols either freshly prepared or stored at −20°C for 15 days were incubated with CC in the absence or presence of dATP. At the end of incubation, samples were fractionated on superose-6 gel filtration column on AKTA FPLC machine. Fractions were collected and 20 µl of different fractions (fraction numbers and M.W. standards are indicated on top) were analyzed by Western blotting for the molecules indicated. Procasp-9, procaspase-9; procasp-3, procaspase-3; CC, cytochrome c.

The preceding experiments have established that, 1) cytosols stored at low temperatures rapidly lose (deoxy)nucleotides including (d)ATP; 2) loss of (deoxy)nucleotides is responsible for the failure of stored cytosols to support CC-initiated caspase activation as exogenous dATP could partially restore the CC-initiated caspase activation in stored cytosols; and 3) dATP does not support complete processing and activation of caspases. To further elucidate why dATP fails to fully support the CC-initiated caspase cleavage/activation in stored cytosols, we carried out gel filtration analysis to evaluate whether stored cytosols are capable of supporting Apaf-1 oligomerization, which is considered to be the rate-limiting step in apoptosome assembly and apoptosome-mediated caspase activation. As shown in Fig. 4, CC alone initiated robust Apaf-1 oligomerization in fresh cytosol. Interestingly, CC alone in cytosolic samples stored at −20°C for 15 days, which retained ∼15% ATP and 36% dATP (Table 1 and 2), induced significant Apaf-1 oligomerization similar to that observed in fresh cytosols and addition of exogenous dATP did not dramatically increase Apaf-1 oligomerization (Fig. 4). Similar results were obtained in gel-filtration experiments using cytosols stored at −80°C for 3 months (not shown). These latter observations suggest that the residual amounts of (d)ATP in the cytosolic samples stored at −20°C for 15 days or at −80°C for 3 months are sufficient for CC-triggered Apaf-1 oligomerization. Altogether, the gel-filtration studies suggest that failure of stored cytosols to fully support the CC-initiated caspase activation is not due to the lack of Apaf-1 oligomerization.

During apoptosome assembly, Apaf-1 oligomerization is accompanied by recruitment and activation of procaspase-9 to generate p35/p37 caspase-9 fragments, which remain in the apoptosome complex and exhibit caspase processing activity (CPA) in the presence of exogenous procaspase-9 and procaspase-3 [23], [24]. Recent studies from the Bratton's group [12] further suggest that auto-processing of caspase-9 starts the molecular timer and is constantly replaced by procaspase-9 until all intracellular procaspase-9 are processed. Processed caspase-9 when present on the apoptosome, recruits and activates procaspase-3 [12]. We found that CC-induced Apaf-1 oligomerization in fresh cytosols was accompanied by the presence of both p35 and p37 caspase-9 fragments in high M.W. fractions with little procaspase-9 (Fig. 4). We also observed prominent p20 caspase-3 but not procaspase-3 in the oligomeric fractions in the CC-activated fresh cytosol (Fig. 4). Cytosols that had been stored at −20°C (Fig. 4) or 4°C (not shown) initiated Apaf-1 oligomerization upon addition of CC or CC/dATP leading to the recruitment of procaspase-9 with the high M.W. fractions, but no processed p35/p37 caspase-9 was observed. Similarly, prominent procaspase-3 was detected in the oligomeric fractions with no cleaved caspase-3 bands in the −20°C cytosols (Fig. 4). These results suggest that in stored cytosols, recruitment of procaspase-9 to the apoptosome is not significantly diminished but the molecular timer [12] is dysfunctional such that recruited procaspase-9 does not undergo auto-processing, and thus, does not dissociate from the apoptosome.

**Figure 5:**
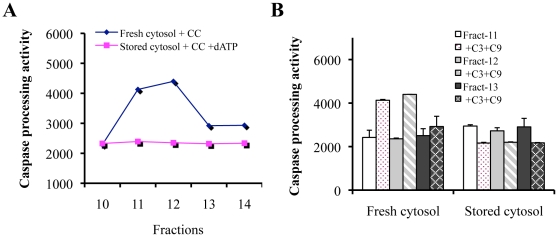
Oligomerized Apaf-1 in stored cytosol fails to activate caspase-3. **A–B**, GM701 cytosols either freshly prepared or stored at −20°C for 15 days were incubated with CC in the absence (for fresh cytosol) or presence (for stored cytosol) of dATP. At the end of incubation, samples were fractionated on superose-6 gel filtration column on AKTA FPLC machine. 20 µl of fractions 10–14 were used for caspase processing activity (CPA) measurement (**A**) by incubating with 100 nM recombinant procaspase-3 (C3) and 100 nM of procaspase-9 (C9) for 90 min at 30°C followed by DEVDase activity measurement. (**B**) CPA measurement in fractions 11–13. Shown are the mean ± S.D (n = 2). CPA activities are presented as arbitrary unit.

Consistent with gel filtration analysis, measurement of caspase-processing activity (CPA) revealed (Fig. 5A–B) that the CC-triggered apoptosome in fresh cytosol (Fig. 4, fractions 10-14) possessed readily detectable CPA whereas apoptosome formed in the −20°C cytosol with the addition of exogenous dATP (Fig. 4, fractions 10–14) did not show significant CPA, indicating non-functional apoptosome.

### Procaspase-9 interacts with Apaf-1 in absence of nucleotides and co-incubation of Apaf-1 with procaspase-9 prior to CC addition inhibits caspase activation

**Figure 6:**
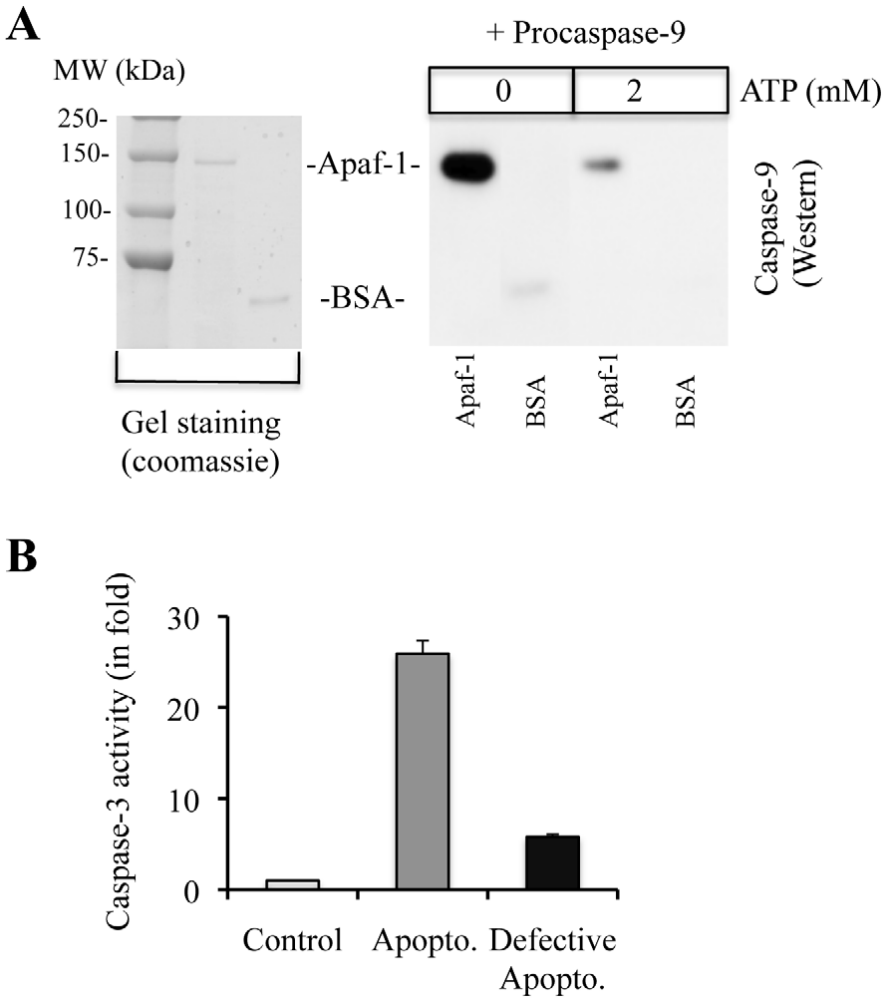
Procaspase-9 interacts with Apaf-1 in the absence of ATP, whereas physiological levels of ATP disrupt Apaf-1-procaspase-9 interaction, and prior incubation of Apaf-1 with procaspase-9 leads to defective apoptosome. **A**, 500 ng of recombinant Apaf-1 or bovine serum albumin (BSA) were immobilized on PVDF membrane. Membrane was then incubated with procaspase-9 (500 ng) in the absence or presence of physiological levels of ATP (2 mM) followed by Western blotting for caspase-9. Left panel represents coomassie stained gel image. **B,** To induce defective apoptosome formation, recombinant Apaf-1 was pre-incubated with procaspase-9 for 30 min at 4°C followed by the addition of procaspase-3, CC, and ATP (200 µM). Caspase-3 activity presented as DEVDase activity. Control, Apaf-1+procaspase-9+procaspase-3; apoptosome, Apaf-1+procaspase-9+procaspase-3+CC+ATP. Apopto, apoptosome.

To understand how procaspase-9 recruitment to the apoptosome leads to defective molecular timer in absence of nucleotides, we performed Far-Western analysis in the presence or absence of physiological levels of ATP (2 mM) using immobilized recombinant Apaf-1. Since intracellular concentration of ATP is in millimolar range and ATP also regulates apoptosome function, we utilized ATP instead of dATP, which generally exists in micromolar range. We observed that procaspase-9 directly interacted with Apaf-1 in the absence of ATP (Fig. 6A). However, the interaction between procaspase-9 and Apaf-1 was drastically reduced in the presence of physiological levels of ATP (Fig. 6A). These results suggest that degradation of nucleotides during storage of cytosols might have promoted the association of procaspase-9 with Apaf-1, which leads to defective apoptosome assembly and function. To further confirm this notion, we incubated recombinant Apaf-1 with procaspase-9 at 4°C for 30 min followed by addition of procaspase-3, CC and ATP to induce apoptosome formation. We observed that CC-induced caspase-9 and caspase-3 activation was inhibited upon pre-incubation of Apaf-1 with procaspase-9 (Fig. 6B).

## Discussion

**Figure 7:**
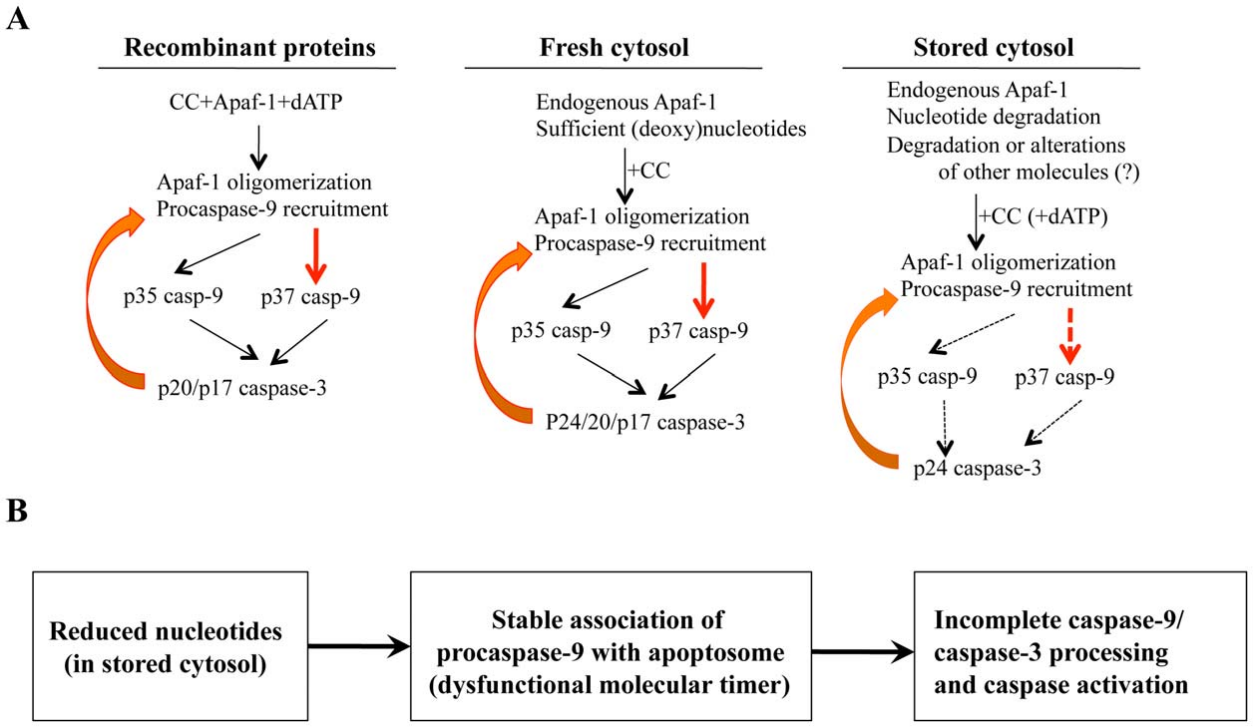
Nucleotide regulates apoptosome-mediated caspase activation. **A**, Summary of cell-free apoptosome reconstitution assays using recombinant proteins, or fresh or stored cytosols. Dashed arrows in ‘Stored cytosol’ indicate attenuated caspase activation. **B,** Defective molecular timer in the absence of nucleotides leads to inefficient caspase activation. See Discussion for details. Casp-9, caspase-9.

Our present work first reveals distinct component requirements when performing cell-free reconstitution of the apoptosome activity under different conditions (Figure 7A). Along the way, we uncover interesting biochemical mechanisms regulating apoptosome activation by nucleotides. In the absence of nucleotides such as in stored cytosols, auto-processing of caspase-9 seems to be inhibited thereby abrogating the molecular timer [12] leading to incomplete caspase-9 processing and inhibition of caspase-3 activation by the apoptosome (Fig. 7B).

Nucleotides such as (d)ATP are absolutely required for apoptosome formation when using recombinant proteins (Fig. 7A, left). In such systems, if procaspase-9 and procaspase-3 are present, both p35 and p37 cleaved caspase-9 bands as well as active p20/p17 caspase-3 fragments can be observed. In contrast, when using freshly purified cytosols to perform cell-free reconstitution experiments, only CC is required to initiate the apoptosome activation due to the presence of sufficient amounts of endogenous nucleotides (Fig. 7A, middle). In such systems, both p35 and p37 caspase-9 and p24/p20/p17 caspase-3 bands are frequently observed (Fig. 1C–D). However, the use of cytosols stored at low temperatures in cell-free reconstitution assays shows defective CC-initiated caspase activation (Fig. 7A, right). Direct measurement of (deoxy)nucleotides demonstrates that storage of cytosols at low temperatures leads to their rapid loss. Strikingly, the degree of nucleotide loss correlates well with the failure of stored cytosols to support the CC-initiated caspase activation. Thus, cytosols that have been stored at 4°C for 5 days, which have completely lost (deoxy)nucleotides, fail to induce caspase activation. However, addition of exogenous (d)ATP partially restores the CC-triggered caspase activation.

In stored cytosols, addition of exogenous dATP did not support full caspase activation evidenced by activity measurement (Fig. 2B–C) and only p35 caspase-9 and mainly p24 caspase-3 are generated in such systems (Fig. 7A, right). These observations suggest that storage of cytosols at low temperatures may lead to not only degradation of (deoxy)nucleotides but also degradation or alteration of some other components important for apoptosome activation. Mechanistic studies demonstrate that cytosols stored at 4°C for 5 days can support robust CC-initiated Apaf-1 oligomerization and procaspase-9 recruitment upon addition of exogenous dATP. These observations suggest that the initial step of apoptosome assembly is normal in stored cytosols, as also supported by the generation of p35 caspase-9 (Fig. 7A, right). Then why can't exogenous dATP support full apoptosome-mediated activation of caspase-9?

We find that although apoptosome formation and caspase-9 recruitment to the apoptosome are not affected, caspase-9 processing is severely compromised. Procaspase-9 was found to be associated with the apoptosome, but a very low level of caspase activity was observed. These findings suggest the requirement of procaspase-9 processing upon recruitment to the apoptosome for full caspase activity. Indeed, Bratton and colleagues [12] have clearly demonstrated that for sustained apoptosome-mediated caspase activation and apoptosis, recruited procaspase-9 must undergo auto-processing (i.e., like a timer) followed by replacement with new procaspase-9. Our findings suggest that in stored cytosols, auto-processing of recruited procaspase-9 does not seem to occur, thus abrogating the molecular timer and leading to sequestration and maintenance of procaspase-9 on the apoptosome (Fig. 7B). Although the precise molecular mechanisms of the stable association of procaspase-9 with the apoptosome in stored cytosols remain to be elucidated, we have observed that procaspase-9 interaction with Apaf-1 is significantly stronger in the absence of nucleotides, suggesting that loss of nucleotides in stored cytosols promotes stable association of procaspase-9 with the Apaf-1 apoptosome leading to dysfunctional molecular timer and inefficient procaspase-9 processing and caspase-3 activation. Indeed, prior incubation of Apaf-1 with procaspase-9 leads to defective caspase activation (Fig. 6). Although the timer mechanism suggests that cells can escape apoptosis in the event of low amounts of apoptosome activation [11], [12], the present findings further support the physiological importance of molecular timer, which may be regulated by (d)ATP.

Cancer cells frequently avert apoptosis by upregulating various prosurvival molecules such as XIAP and Hsp70, which promote cell survival by inhibiting caspase activation [24], [25], [26], [27], [28], [29], [30]. For example, XIAP binds with active caspase-9 and caspase-3 to inhibit the caspase cascade, and thus, apoptosis. Similarly, Hsp70 has been shown to abrogate apoptosome formation and function. It is also important to note that Hsp70 plays an important role in nucleotide exchange and is essential for functional apoptosome assembly [31]. It is possible that in the absence of (d)ATP, Hsp70 may associate with the apoptosome, and is not available for performing nucleotide exchange during the complex formation. Therefore, in the absence of nucleotide exchange, the recruited procaspase-9 is not active and continues to associate with the apoptosome. During stress and apoptotic stimulation, when mitochondria-mediated ATP generation is compromised and the levels of nucleotides are reduced [16], procaspase-9 may stably associate with Apaf-1 apoptosome inhibiting caspase activation and apoptosis. Therefore, these findings further provide evidence that nucleotide pools, especially the ATP levels, play an important role in regulating apoptotic cell death by targeting apoptosome.

## Materials and Methods

### Cells and reagents

GM701 (immortalized human fibroblasts) cells kindly provided by Dr. M. King (Thomas Jefferson University) were cultured in Dulbecco's Minimum Essential Medium (DMEM; Gibco Grand Island, NY) supplemented with 10% heat-inactivated fetal bovine serum (FBS) and antibiotics. Human prostate cancer cells, PC3, LNCaP, Du145, and PPC1 were purchased from ATCC (Rockville, MD) and cultured in RPMI 1640 supplemented with 10% FBS. MDA-MB231 (breast cancer) and HeLa (cervical cancer) cells were obtained from ATCC and cultured in MEM with 10% FBS. SKOV-3 (ovarian cancer) obtained from ATCC and cultured in McCoy's 5a medium with 10% FBS. K562 (leukemia), C2F3 (myofibroblasts) were procured from ATCC and cultured in RPMI with 10% FBS. HCT116 (colon cancer) cells were provided by Dr. B. Vogelstein and cultured in DMEM supplemented with 10% FBS. NHDF-neo (normal human dermal fibroblasts- neonatal), HMEC (human mammary epithelial cells), and NHP (normal human prostate) cells were purchased from Lonza (Walkersville, MD USA) and cultured in company recommended medium [16], [18], [32], [33], [34], [35]. Primary antibodies (Ab) used in this study were: rabbit (Rb) pAb to caspase-3 (Biomol, Cat # SA320); mAbs to CC (BD PharMingen, Cat # 556433), Rb pAbs to Apaf-1 (BD PharMingen, Cat # 559683); and Rb pAb to caspase-9 (Chemicon, Cat # AB16970), mAb to actin (MP Biomedicals, Cat # 69100). All secondary antibodies, and enhanced chemiluminescence (ECL) reagents were from GE Healthcare. DEVD-AFC (Cat # P-409), and LEHD-AFC (Cat # P-445) were bought from Biomol. All other chemicals were purchased from Sigma (St. Louis, MO) unless specified otherwise.

### Subcellular fractionation and Western blotting

The cytosol and mitochondria were purified as described previously [16], [18], [32], [33], [34], [35]. Various types of cells were harvested, washed twice in ice-cold PBS, and resuspended in homogenizing buffer (20 mM HEPES-KOH, pH 7.5, 10 mM KCl, 1.5 mM MgCl_2_, 1 mM sodium EDTA, 1 mM sodium EGTA and 1 mM dithiothreitol) containing 250 mM sucrose and a mixture of protease inhibitors (1 mM PMSF, 1% aprotinin, 1 mM leupeptin, 1 µg/ml pepstatin A and 1 µg/ml chymostatin). After 30 min incubation on ice, cells were homogenized using a glass Pyrex homogenizer (type A pestle, 140 strokes) and centrifuged twice at 1000 g for 5 min at 4°C. The resulting supernatant was centrifuged at 10,000 g for 20 min to obtain mitochondria as pellet. Supernatant was further subjected to centrifugation at 100,000 g for 1 h to obtain cytosolic fraction (i.e., S100 fraction). Protein concentration was determined by Micro-BCA kit (Pierce, Rockford, IL).

For Western blotting, samples were loaded on SDS polyacrylamide gel. After gel electrophoresis and protein transfer, the membrane was probed or reprobed, after stripping, with various primary and corresponding secondary antibodies. Western blotting was performed using ECL as previously described [16], [18], [32], [33], [34], [35].

### Far-Western analysis

Far Western analysis was performed as described previously [16]. Apaf-1 or BSA was immobilized on PVDF membrane through gel electrophoresis. The membrane was blocked with 5% nonfat dry milk overnight and then incubated with either procaspase-9 alone or procaspase-9 in the presence of ATP and 1% BSA for 1 hour in Buffer A (20 mM HEPES-KOH, pH 7.5, 10 mM KCl, 1.5 mM MgCl_2_, and 1 mM each of EDTA, EGTA, and DTT) at room temperature. After thorough washing with buffer A, the membrane was probed for caspase-9.

### Caspase activity measurements

Caspase-9 (i.e., LEHDase) or caspase-3 (i.e., DEVDase) activities were determined as described previously [16], [18], [32], [33], [34], [35]. Briefly, reconstituted cytosolic proteins were added to the caspase reaction mixture containing 30 µM fluorogenic peptide substrates, DEVD-AFC (for caspase-3) or LEHD-AFC (for caspase-9), 50 mM of HEPES, pH 7.4, 10% glycerol, 0.1% CHAPS, 100 mM NaC1, 1 mM EDTA, and 10 mM DTT, in a total volume of 100 µl and incubated at 37°C for 90 min. Production of 7-amino-4-trifluoromethyl-coumarin (AFC) was monitored on spectrofluorimeter using excitation wavelength 400 nm and emission wavelength 505 nm. The results were presented as fold activation over the control.

### Cell-free reconstitution

All cell-free reactions were performed in homogenizing buffer (20 mM HEPES-KOH, pH 7.5, 10 mM KCl, 1.5 mM MgCl_2_, 1 mM sodium EDTA, 1 mM sodium EGTA and 1 mM dithiothreitol) containing 250 mM sucrose and a mixture of protease inhibitors in a total volume of 100 µl [16], [18]. Briefly, cytosols were activated by adding bovine CC (15 µg/ml) with or without dATP and incubated for 150 min at 37°C. In some experiments, cytosols were first stored at different temperatures and then activated by bovine CC with or without dATP. In some reconstitution experiments, cytosols were incubated with recombinant Apaf-1, procaspase-9, and procaspase-3 alone or in different combinations as indicated in the Figure legends. After incubation, samples were either used for substrate cleavage assays for caspase-9 (LEHDase) and caspase-3 (DEVDase) or for caspase processing by Western blotting.

### Analysis of apoptosome complexes

Control, CC-activated, or CC/dATP-activated cytosols were fractionated by size-exclusion chromatography using Superose 6 column (GE Healthcare) calibrated with thyroglobulin (669 kDa), ferritin (440 kDa), aldolase (158 kDa), bovine serum albumin (67 kDa), ovalbumin (43 kDa), chymotrypsinogen A (25 kDa), and ribonuclease A (13.7 kDa) (all obtained from GE Healthcare). Apoptosome complexes (∼700 kDa) were eluted using column buffer (20 mM HEPES, 0.1% (w/v) CHAPS, 5 mM DTT, 5% (w/v) sucrose pH 7.0) supplemented with 50 mM NaCl. Different fractions were then analyzed by Western blotting for changes in the distribution of Apaf-1, processing of caspases [3], [5], [16]. For caspase processing activity, 20 µl of fractions 11–14 were incubated with 100 nM of recombinant procaspase-9 and procaspase-3 for 90 min followed by DEVDase activity measurement.

### Purification of recombinant Apaf-1, procaspase-9, and procaspase-3

Apaf-1, procaspase-9, and procaspase-3 were cloned into pFastbacH, a modified version of pFastbac with a C-terminal 6 histidine tag, expressed in insect cells (Sf9) and purified on Nickel agarose as previously described [12], [16], [36], [37].

### Determination of intracellular dNTP pools

The levels of dNTP from various cytosols were determined as described previously [16], [38]. Briefly, nucleotides were extracted from cytosolic samples using 60% methanol. The DNA polymerase assay was utilized to quantify dNTPs in the cell extracts. The Klenow fragment of DNA polymerase I lacking exonuclease activity (USB, Cleveland, OH) was used to start a reaction in a mixture that contained 100 mM HEPES buffer (pH 7.3), 10 mM MgCl2, 7.5 µg BSA, and synthetic oligonucleotides of defined sequences as templates annealed to a primer, [^3^H]dTTP or [^3^H]dATP and either standard dNTP or the extract from 1 or 2×10^6^ cells. Reactants were incubated for 1 h and applied to filter discs. After washing, the radioactivity on the disks was determined by liquid scintillation counting and compared with that in the standard dNTP samples [16], [38].

### Measurement of nucleotide triphosphates by HPLC

To measure the NTPs levels, nucleotides were extracted using perchloric acid. The nucleotide extracts were neutralized with KOH [39]. The neutralized extracts were applied to an anion-exchange Partisil-10 SAX column and eluted at a flow rate of 1.5 ml/min with a 50-min concave gradient (curve 7; Waters 600E System Controller; Waters Corp.) from 60% 0.005 M NH_4_H_2_PO_4_ (pH 2.8) and 40% 0.75 M NH_4_H_2_PO_4_ (pH 3.6) to 100% 0.75 M NH_4_H_2_PO_4_ (pH 3.6). The column elute was monitored by UV absorption at 256 nm, and the nucleoside triphosphates were quantified by electronic integration with reference to external standards. The lower limit of sensitivity of this assay was 10 pmol in an extract of 5×10^6^ cells corresponding to a cellular concentration of 1 µM [16], [38].
